# Suicidality in psychiatric emergency department situations during the first and the second wave of COVID-19 pandemic

**DOI:** 10.1007/s00406-022-01486-6

**Published:** 2022-09-07

**Authors:** Yann David Kippe, Maia Adam, Anna Finck, James Kenneth Moran, Meryam Schouler-Ocak, Felix Bermpohl, Stefan Gutwinski, Thomas Goldschmidt

**Affiliations:** Psychiatrische Universitätsklinik der Charité Im St. Hedwig Krankenhaus, Große Hamburger Str. 5-11, 10115 Berlin, Germany

**Keywords:** Suicidality, COVID-19, Psychiatric emergency department, Borderline personality disorder, Substance use disorder

## Abstract

Psychiatric patients are prone to mental health deterioration during the Covid-19 pandemic. Little is known about suicidality in psychiatric patients during the Covid-19 pandemic. This study is a retrospective chart review of psychiatric emergency department (pED) presentations with present or absent suicidality (5634 pED attendances, 4110 patients) in an academic pED in Berlin, Germany. Poisson regression analysis was performed on the effect of Covid-19 period on suicidality (suicidal ideation (SI), suicide plans (SP) or suicide attempt (SA)) during the first (3/2/2020–5/24/2020 “first-wave”) and second (9/15/2020–3/1/2021 “second-wave”) wave of the Covid-19 pandemic compared to the same periods one year earlier. During the first-wave the number of pED visits per person with SI, SP and SA was higher compared to one year earlier (SI RR = 1.614; *p* = 0.016; SP RR = 2.900; *p* = 0.004; SA RR = 9.862; *p* = 0.003). SI and SP were predicted by interaction between substance use disorder (SUD) and second-wave (SI RR = 1.305, *p* = 0.043; SP RR = 1.645, *p* = 0.018), SA was predicted by interaction between borderline personality disorder (BPD) and second-wave (RR = 7.128; *p* = 0.012). Suicidality increased during the first-wave of Covid-19 pandemic in our sample. In the second-wave this was found in patients with SUD and BPD. These patients may be at particular risk of suicidality during the Covid-19 pandemic.

**Supplementary Information:**

The online version contains supplementary material available at 10.1007/s00406-022-01486-6.

## Introduction

The Covid-19 pandemic poses a major challenge to societies and individuals worldwide. Early warnings about adverse mental health consequences are backed up by a growing body of evidence, as studies have found elevated levels of depression and anxiety symptoms in population-based cohorts [1, 2]. Patients with pre-existing mental disorders appear to be especially vulnerable to negative outcomes in terms of mental health during the Covid-19 pandemic [3–5].

One aspect of particular importance when assessing mental health outcomes is suicidality, a complex and multi-faceted phenomenon [6]. Elevated rates of suicidality have been linked to unemployment [7], homelessness [8], uncertain economic situation [9], loneliness [10] and to stressful life events [11], which have increased during the Covid-19 pandemic [12]. A study focusing on suicidal ideation in the general population of the UK found elevated levels, which increased over the first three infection waves of the pandemic [13]. Yet, studies examining suicide death rates have found no overall difference or a decline compared to pre-pandemic times [14–16], only one study in Germany reported an increase of suicidality in the subgroup of elderly women [17].

Another method of investigating suicidality is to examine suicidal psychiatric emergency department (pED) attendances [18]. So far, this approach has produced heterogenous results, ranging from an absolute increase in numbers of attendances with suicidal behavior [19] or proportional increase of attendances with suicidal ideation [20] to studies showing no difference regarding suicidal ideation or attempts [21, 22] or even observing declining numbers of presentations [23, 24], with suicidality in pEDs during the Covid-19 pandemic. Some have observed upward rebounds of suicidality after lifting of lockdown and contact restriction measures [25–27]. The pED studies, so far, display a great heterogeneity in terms of observed time periods, study location, co-occurring Covid-19 infection rates and government restrictions. To date, only few studies have focused on clinical and demographic features of pED presentations with suicidality during the first-wave of Covid-19. One reporting a pandemic-driven increase of suicidality in homeless patients in Boston, USA [28] and another reporting an increase in suicidal ideation in patients with substance use disorder in Hannover, Germany [29].

The current study relies on clinical documentation of pED presentations of an academic pED in Berlin, Germany and aims to better describe this particularly vulnerable cohort of patients presenting to the pED with suicidality (i.e. with current suicidal ideation (SI), suicide plans (SP) and/or suicide attempt (SA) directly before presenting to the pED). For the two first Covid-19 waves in Germany, we were interested in whether there was an effect on suicidality in patients presenting to an pED that could be related to the Covid-19 period independently of major risk factors and comorbidities for suicidality, such as psychiatric diagnoses [30], gender [31], age [32] and homelessness [28]. Furthermore, we were interested in whether the Covid-19 period would affect diagnostic categories known to be at particular risk of suicidality differently (interaction effect). For this, we considered borderline personality disorder (BPD), substance use disorders (SUD), depressive disorders (DD) and schizophrenia and psychotic disorders (SPD) based on Nock et al. [30]. Poisson regression models were used to test for the Covid-19 period and the interaction effect. Additionally, data on the occurrence of suicidality across all assessed diagnostic categories is provided at the event level.

## Methods

### Study design

We conducted a retrospective analysis of clinical records of all patients attending the psychiatric clinic of Charité University Berlin at St. Hedwig Hospital (SHK) in Berlin during the first (3/2/2020 – 5/24/2020 “first-wave”) and the second (9/15/2020 – 3/1/2021 “second-wave”) wave of the Covid-19 pandemic in Germany. For comparison, data from the same time periods one year earlier were obtained (“control period”). The study was approved by the local ethics committee (Charité University, Berlin: EA110/20).

The observed Covid-19 periods cover different extents of social distancing and lockdown measures (cf. dotted lines in Fig. 2). The beginning of the first-wave (3/2/2020) is marked by the date of the first publicly known Covid-19 case in Berlin [33]; it spans until the date of the curve reaching the bottom number of newly registered cases of Covid-19 infections in Berlin (5/24/2020), marking the end of the first-wave [34]. The second-wave begins with continuously rising 7-day incidences [34] (9/15/2020) and ends with first lifting of lockdown measures in Berlin [35] (3/1/2021).

The psychiatric department at SHK consists of an emergency department and seven psychiatric care units for inpatient treatment. It serves as the psychiatric hospital for the districts Moabit, Tiergarten and Wedding in Berlin, with a catchment area of approximately 327.000 people. The SHK psychiatric department is obliged to provide inpatient treatment for patients living within the above-mentioned districts and having an indication for hospital admission, whereas patients who live in other districts of Berlin are usually redirected to the psychiatric department of their district when hospital admission is required.

Based on a modified version of the Covid-19 risk group classification of Robert-Koch-Institute [36], we defined a Covid-19 risk group being at risk of severe Covid-19 pneumonia, utilizing documentation of somatic medical conditions. All considered criteria for the Covid-19 risk group can be found in supplementary material S1.

We analyzed pED presentations both on a patient-level with sociodemographic and clinical characteristics (patient-per-period format with calculated count variables for the number of suicidal ED visits per person and period) and on an event level, giving information on absolute numbers of pED presentations, suicidality rates and suicidality across diagnostic categories. At patient-level, risk factors and diagnoses were documented if they occurred in at least one pED presentation; sociodemographic variables were obtained from the first case of a patient in the corresponding period. Patient-level data was analyzed utilizing Poisson regression, exploiting the ability to demonstrate how certain features influence the number of pED presentations with suicidality on an individual level. This provides quantifiable effect sizes, expressed as rate ratios with 95% confidence intervals. Also, repeated attendances with suicidality are considered. I.e., the Poisson regression model helps to answer the question of whether the respective period (first-wave or second-wave) contributes to a relevant extent to the number of pED presentations with suicidality per patient or if this number can better be explained by other risk factors and comorbidities.

Diagnoses were grouped into the following categories: organic mental disorders (OMD), substance use disorders (SUD), schizophrenia and psychotic disorders (SPD), bipolar and manic disorders (BMD), depressive disorders (DD), neurotic, somatoform, and stress related disorders (NSD) and personality disorders (PD). A list of included ICD-10 diagnoses for each category can be found in supplementary material S2. We considered the principal diagnosis and all secondary diagnoses for the classification, as the principal diagnosis was not always clearly distinguishable.

**Fig. 1:**
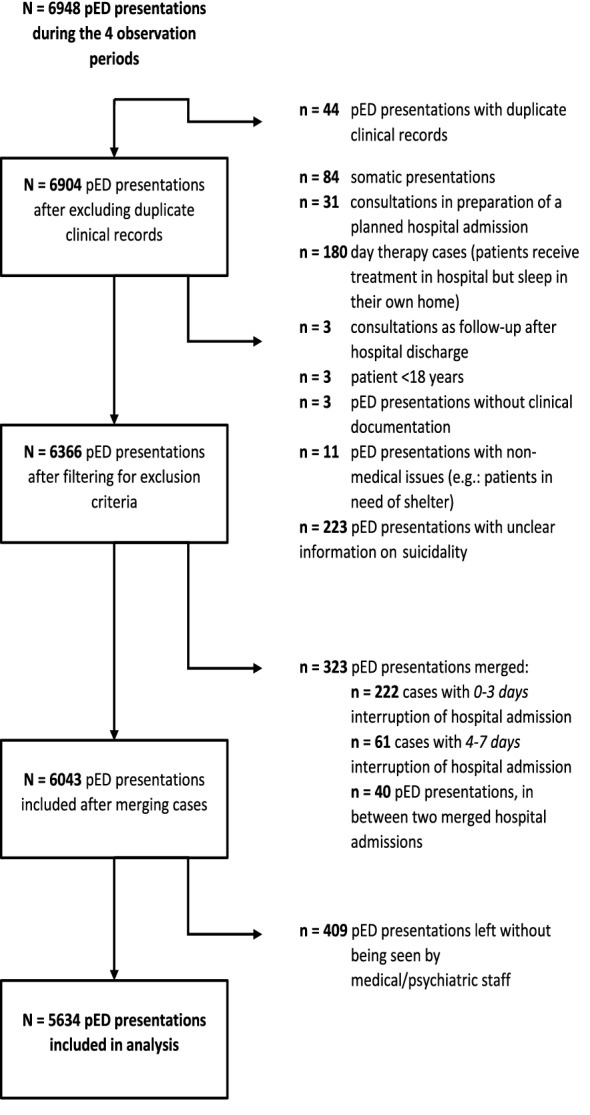
Algorithm of excluded cases. Overview of the exclusion-procedure of pED presentations in form of a flow chart. Boxes on the left indicate the number of pED presentations that remained after each step. On the right, excluded pED presentations are listed by the reason for their exclusion. Abbreviations used: pED = psychiatric emergency department

Cases were excluded if they concerned a day therapy unit (which were shut down during the beginning of the pandemic), if they left without being seen by a psychiatrist, if no documentation was available on suicidality or if no psychiatric F-diagnosis according to the International Statistical Classification of Diseases and Related Health Problems, 10th revision (ICD-10) was documented. For transformation of our data from event- to patient-based format, three additional cases had to be excluded because of unknown identity. Considering high-frequent attenders and the possible bias this group would impose, we merged pED presentations with subsequent hospital admissions if they were separated by less than 3 days. If cases were separated by 4–7 days, they were only merged if discharge was due to somatic complications or against documented advise of medical staff. An overview of all excluded and merged cases is depicted in Fig. 1.

### Statistical analysis

For the description of categorical variables, absolute numbers and rates are reported. Percentages are compared using Chi^2^ test and, in cases where this was not applicable, Fisher’s exact test. Quantitative variables were tested for normal distribution utilizing the Kolmogorov–Smirnov-Test and via graphic examination of the Q-Q-Plot. We used Mann–Whitney-*U*-Test for metric variables that were not normal distributed.

Count variables were tested for Poisson-distribution using the Kolmogorov–Smirnov-Test. All count variables are Poisson-distributed. We fitted Poisson regression models for each suicidality outcome and wave individually to assess the effect of the first two waves of the Covid-19 pandemic separately, whilst controlling for comorbidities and risk factors. We chose to control for diagnostic categories and for risk factors found in literature regarding the subject, limiting ourselves to those that were well documented in our primary data. We thus ended up controlling for gender [31], age [32], living in a residential psychiatric therapeutic environment [37], being homeless [28] and belonging to a Covid-19 risk group [38]. Since suicidality and self-harming behaviors are one of the key traits to BPD [39, 40], we decided to further discriminate between BPD and other PD.

BPD [30, 39, 40], SUD [30], DD [30, 41, 42] and SPD [30, 43] are diagnostic groups known to be associated with elevated risk of suicidal behavior, thus we decided to include time dependent interaction effects between these groups and Covid-19 period in our Poisson regression models. Results from Poisson regression models are presented as rate ratios (RR) with 95% confidence intervals (95% CI) and are tested for significance using the Wald-Chi^2^ test. The overall significance level was set to *p* < 0.05. For all Poisson regression models, alternative negative binomial regression models were fitted and compared regarding model fit using the Akaike Information Criterion (AIC, see supplementary material S3). Poisson models showed superior model fit in five out of six models; in one case the difference in favor of the negative binomial model was only minor, so we continued with Poisson models for better comparability. Results of the negative binomial regression models are displayed as a sensitivity analysis in supplementary material S4. All statistical analyses were performed using the SPSS statistical package, version 27.0, IBM Corporation (2020); figures were created using MS Excel 365, Microsoft Corporation (2020).

## Results

A total of *N* = 6948 pED records were documented during the four observed time periods. After applying exclusion criteria (cf. Figure 1), a total of *N* = 5634 pED presentations were included in our analysis (Table 1), with the total number of patients being *N* = 4110. For description of demographic and clinical characteristics of patients presenting to the pED, see supplementary material S3.

**Table 1:** Characterization of pED presentations

	Control period (2019)	First-wave	Difference	*p*-value	Control period (2019/2020)	Second-wave	Difference	*p*-value
*N* total number of pED presentations	922	797	– 13.6%	–	1941	1974	+ 1.7%	–
Mean pED presentations per week (SD)	76.8 (6.85)	66.4 (11.37)	– 13.5%	**0.013**	80.9 (6.86)	82.3 (9.57)	+ 1.7%	0.570
Median age	39 years	39 years	± 0 years	0.159	39 years	39 years	± 0 years	0.988
Female gender (%)	388 (42.1%)	305 (38.%)	–21.4%	0.108	772 (39.8%)	780 (39.5%)	+ 1.0%	0.878
Covid-19 positive	–	0 (0.0%)	–	–	–	13 (0.7%)	–	–
Suicidality								
Suicidal ideation (SI)	204 (22.1%)	220 (27.6%)	+ 7.8%	**0.009**	510 (26.3%)	483 (24.5%)	– 5.3%	0.191
Suicide plans (SP)	60 (6.5%)	83 (10.5%)	+ 38.2%	**0.003**	215 (11.1%)	213 (10.8%)	– 0.9%	0.752
Suicide attempt (SA)	19 (2.1%)	36 (4.5%)	+ 89.5%	**0.004**	79 (4.1%)	80 (4.1%)	+ 1.3%	0.970

Comparison of demographic and clinical characteristics of pED presentations (event level) in corresponding time periods of the first-wave (on the left) and the second-wave (on the right). Difference is the change of case numbers in the Covid-19 period compared to the corresponding control period in percentages. *P* values (bold = significant to a level of *p* ≤ 0.05) are derived from chi^2^-tests, except for "median age", which were tested using the Mann–Whitney-*U*-test. "Covid-19 positive" includes all patients tested positive for Covid-19 at admission or during hospital treatment. Abbreviations used: pED = psychiatric emergency department; *N* = patient numbers; Covid-19 = coronavirus disease 2019; SD = standard deviation

### Poisson regression results (patient-per-period level)

Poisson regression models estimated the first-wave to be an independent risk factor for all three suicidality outcomes in our sample (SI RR: 1.614; 95% CI 1.093–2.382; *p* = 0.016; SP RR: 2.900; 95% CI 1.415–5.943; *p* = 0.004; SA RR: 9.862; 95% CI 2.171–44.806; *p* = 0.003), whereas no effect was found regarding the second-wave (Table 2).

**Table 2:** Poisson models estimating effects of the first-wave and second-wave of Covid-19 on suicidal ideation (SI), suicide plans (SP) and suicide attempts (SA)

Suicidal ideation (SI)	First-wave	*p*-value	Second-wave	*p*-value
	RateRatio (95% CI)		RateRatio (95% CI)	
Covid-19 (vs. control)	1.614 (1.093–2.382)	**0.016**	0.993 (0.781–1.262)	0.953
Interaction effects (time dependent)				
Borderline personality disorder by Covid-19	1.362 (0.815–2.276)	0.239	0.822 (0.581–1.165)	0.271
Substance use disorders by Covid-19	0.753 (0.504–1.125)	0.166	1.305 (1.008–1.689)	**0.043**
Depressive disorders by Covid-19	0.857 (0.541–1.358)	0.512	1.011 (0.757–1.350)	0.943
Schizophrenia and psychotic disorders by Covid-19	1.031 (0.618–1.720)	0.906	0.750 (0.544–1.034)	0.079
Diagnostic categories (time independent)				
Organic mental disorders	0.761 (0.406–1.427)	0.395	1.015 (0.706–1.460)	0.936
Substance use disorders	1.482 (1.097–2.001)	**0.010**	1.226 (1.017–1.478)	**0.033**
Schizophrenia and psychotic disorders	0.851 (0.575–1.257)	0.417	1.294 (1.023–1.636)	**0.032**
Bipolar and manic disorders	1.015 (0.630–1.635)	0.953	1.411 (1.044–1.907)	**0.025**
Depressive disorders	2.187 (1.585–3.019)	** < 0.001**	2.138 (1.744–2.622)	** < 0.001**
Neurotic, somatoform and stress related disorders	1.379 (1.083–1.756)	**0.009**	1.558 (1.338–1.813)	** < 0.001**
Borderline personality disorder	2.375 (1.616–3.491)	** < 0.001**	2.908 (2.262–3.738)	** < 0.001**
Other personality disorders	1.779 (1.222–2.590)	**0.003**	2.016 (1.602–2.538)	** < 0.001**
Sociodemographic risk factors (time independent)				
Male	1.017 (0.825–1.252)	0.877	1.145 (0.996–1.316)	0.056
Age	0.991 (0.983–0.998)	**0.018**	0.991 (0.986–0.997)	**0.001**
Living in residential therapeutic environment	1.474 (0.960–2.264)	0.076	1.350 (1.080–1.688)	**0.008**
Homeless	1.431 (1.060–1.933)	**0.019**	1.529 (1.270–1.840)	** < 0.001**
Covid-19 risk group	1.235 (0.959–1.590)	0.102	1.437 (1.206–1.712)	** < 0.001**
Suicide plans (SP)				
Covid-19 (vs. control)	2.900 (1.415—5.943)	**0.004**	0.881 (0.599—1.295)	0.519
Interaction effects (time dependent)				
Borderline personality disorder by Covid-19	1.628 (0.691–4.287)	0.323	1.638 (0.896–2.994)	0.109
Substance use disorders by Covid-19	0.520 (0.249–1.087)	0.082	1.645 (1.088–2.487)	**0.018**
Depressive disorders by Covid-19	0.769 (0.353–1.672)	0.507	0.804 (0.509–1.272)	0.351
Schizophrenia and psychotic disorders by Covid-19	0.788 (0.286–2.174)	0.646	1.034 (0.610–1.752)	0.901
Diagnostic categories (time independent)				
Organic mental disorders	0.308 (0.073–1.300)	0.109	0.990 (0.567–1.731)	0.973
Substance use disorders	1.692 (0.943—3.036)	0.078	1.168 (0.861–1.585)	0.319
Schizophrenia and psychotic disorders	0.659 (0.297–1.460)	0.304	0.928 (0.617–1.396)	0.720
Bipolar and manic disorders	1.550 (0.783–3.072)	0.209	1.286 (0.787–2.100)	0.315
Depressive disorders	2.635 (1.445–4.805)	**0.002**	2.386 (1.731–3.290)	** < 0.001**
Neurotic, somatoform and stress related disorders	1.019 (0.647–1.606)	0.934	1.551 (1.217–1.977)	** < 0.001**
Borderline personality disorder	2.012 (0.914–4.428)	0.083	1.706 (1.050–2.772)	**0.031**
Other personality disorders	2.547 (1.434–4.522)	**0.001**	1.911 (1.327–2.751)	** < 0.001**
Sociodemographic risk factors (time independent)				
Male	1.482 (1.011–2.170)	0.044	1.392 (1.110–1.745)	**0.004**
Age	0.995 (0.982–1.009)	0.503	0.997 (0.988–1.005)	0.424
Living in residential therapeutic environment	1.100 (0.477–2.536)	0.824	1.084 (0.732–1.606)	0.686
Homeless	1.597 (0.959–2.669)	0.072	1.577 (1.187–2.096)	**0.002**
Covid-19 risk group	1.507 (0.981–2.316)	0.061	1.270 (0.962–1.677)	0.092
Suicide attempt (SA)				
Covid-19 (vs. control)	9.862 (2.171–44.806)	**0.003**	1.146 (0.590–2.223)	0.688
Interaction effects (time dependent)				
Borderline personality disorder by Covid-19	1.307 (0.304–5.616)	0.719	7.128 (1.528–33.258)	**0.012**
Substance use disorders by Covid-19	0.391 (0.105–1.456)	0.162	1.279 (0.624–2.619)	0.501
Depressive disorders by Covid-19	0.168 (0.038–0.739)	**0.018**	0.663 (0.293–1.502)	0.325
Schizophrenia and psychotic disorders by Covid-19	0.488 (0.091–2.621)	0.403	0.345 (0.124–0.961)	**0.042**
Diagnostic categories (time independent)				
Organic mental disorders	0.805 (0.162–4.000)	0.791	1.180 (0.483–2.881)	0.716
Substance use disorders	1.694 (0.582–4.935)	0.334	1.253 (0.728–2.156)	0.415
Schizophrenia and psychotic disorders	1.690 (0.413–6.909)	0.465	1.023 (0.528–1.981)	0.947
Bipolar and manic disorders	0.491 (0.065–3.711)	0.490	0.354 (0.086–1.460)	0.151
Depressive disorders	9.171 (2.921–28.790)	** < 0.001**	1.999 (1.118–3.576)	**0.020**
Neurotic, somatoform and stress related disorders	0.383 (0.131–1.115)	0.078	1.186 (0.766–1.846)	0.444
Borderline personality disorder	6.374 (1.879–21.620)	**0.003**	0.401 (0.096–1.678)	0.211
Other personality disorders	3.219 (1.202–8.618)	**0.020**	1.420 (0.686–2.940)	0.345
Sociodemographic risk factors (time independent)				
Male	2.443 (1.222–4.885)	**0.012**	0.974 (0.663–1.433)	0.895
Age	1.009 (0.985–1.034)	0.473	0.994 (0.979–1.009)	0.410
Living in residential therapeutic environment	0.554 (0.075–4.123)	0.564	0.978 (0.473–2.023)	0.952
Homeless	1.425 (0.574–3.536)	0.445	1.157 (0.666–2.010)	0.604
Covid-19 risk group	1.240 (0.583–2.638)	0.576	1.105 (0.667–1.831)	0.699

Results from the Poisson regression models estimating effects of "Covid-19 period" vs. "Control period" on the number of pED presentations with suicidality (SI, SP and SA) per patient. Rate ratios greater than 1 indicate that a factor is increasing the number of presentations per person with suicidality. Rate ratios below 1 indicate that a factor is decreasing the number of presentations per person with suicidality. bold = significant to a level of *p* ≤ 0.05; Covid-19 risk group is defined by a modified classification of the Robert-Koch-Institute, criteria can be found in supplementary material S2.3. "Living in a residential therapeutic environment" is composed of patients living in therapeutic residential groups and living alone with psychiatric and/or social assistance. Abbreviations used: 95% CI = 95% confidence interval; Covid-19 = coronavirus disease 2019

During the first-wave, time dependent interaction effects showed decreased risk of a pED attendance after SA in patients with DD (RR: 0.168; 95% CI: 0.038–0.739; *p* = 0.018). During the second-wave, time dependent interactions showed increased risk in patients with SUD for SI (RR: 1.305; 95% CI: 1.008–1.689; *p* = 0.016) and for SP (RR: 1.645; 95% CI: 1.088–2.487; *p* = 0.018). Interaction effect between BPD and second-wave showed elevated risk for a pED presentation after SA (RR: 7.128; 95% CI: 1.528–33.258; *p* = 0.012), whereas interaction between SPD and second-wave was associated with reduced risk for pED presentation after SA (RR: 0.345; 95% CI: 0.124–0.961; *p* = 0.042).

During the first-wave and its control-period psychiatric conditions that were time independently most strongly associated with suicidal outcomes were DD (SI RR: 2.187, *p* < 0.001; SP RR: 2.635, *p* = 0.002; SA RR: 9.171, *p* < 0.001) and BPD (SI RR: 2.375, *p* < 0.001; SP: no elevated risk; SA RR: 6.374, *p* = 0.003) and compared to BPD to a lesser extent other PD (Table 1). Male gender showed an increased risk for SA in the model for the first-wave and its control period (SA RR: 2.443, *p* = 0.012).

During the second-wave and its control period, DD (SI RR: 2.138, *p* < 0.001; SP RR: 2.386, *p* < 0.001; SA RR: 1.999, *p* = 0.020) and BPD (SI RR: 2.908, *p* < 0.001; SP RR: 1.706, *p* = 0.031, SA: no elevated risk) were time independently most strongly associated with suicidal outcomes and compared to BPD to a similar extent other PD (Table 1).

Other time independent regressors, that were significantly associated with elevated risk for SI and SP during the first-wave and its control period and/or the second-wave and its control period were NSD, SUD, BMD, SPD, being homeless, belonging to Covid-19 risk group, male gender and living in a residential psychiatric therapeutic environment. Older age had a protective effect for SI.

Table 1 displays the respective rate ratios, confidence intervals and p-values of all regressors used in the models.

### Comparison of the two first Covid-19 waves and their respective control periods regarding pED presentations (event level)

The mean rate of weekly pED presentations decreased by 15.0% (2019: 76.8 (SD = 6.85), 2020: 66.4 (SD = 11.37), *p* = 0.013, Cohen’s *d* = –1.110) during the first-wave compared to its control period but did not change significantly during the second-wave compared to its control period (Table 1).

**Fig. 2:**
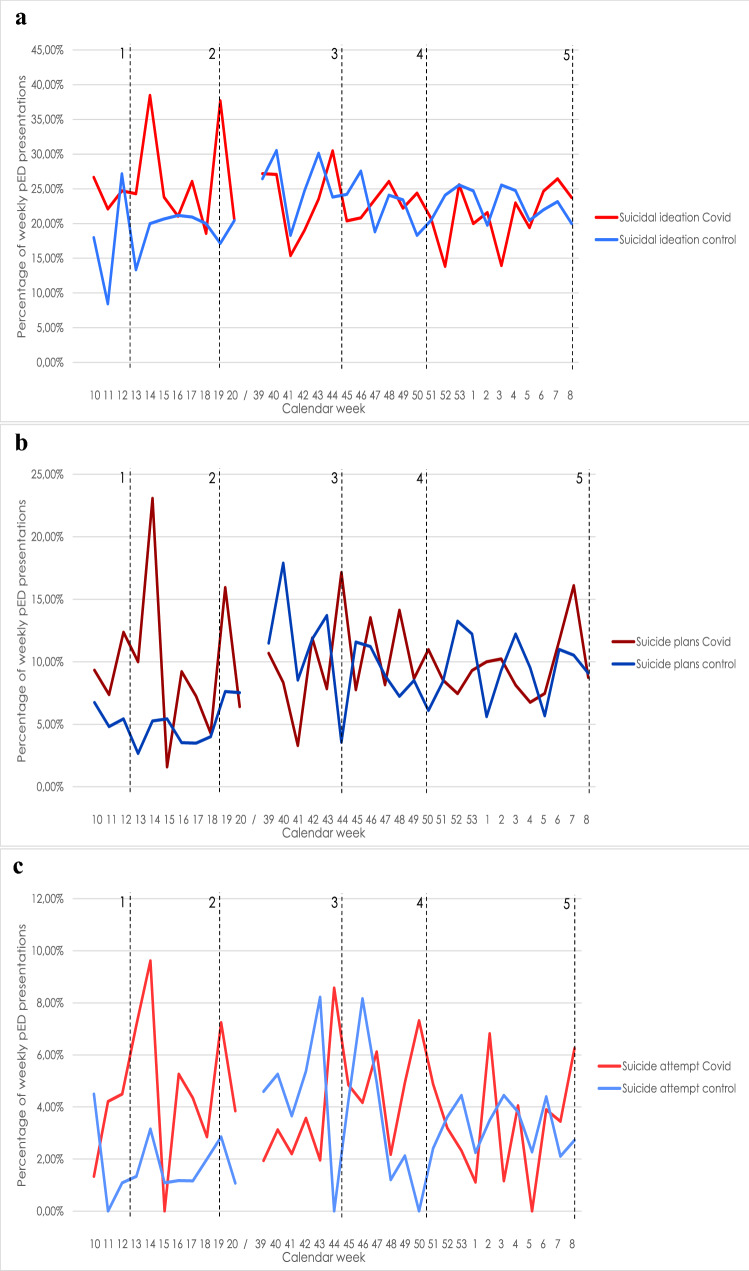
pED presentations with suicidality. Relative number of pED presentations with suicidality out of all pED presentations per week in percentages. Red lines indicate values for Covid-19 periods in 2020/2021, blue lines indicate values for control periods 2019/2020. Calendar week indicates weeks in the years 2020 and 2021. As 2019 had only 52 weeks, but 2020 had 53 weeks, the corresponding week for 53/2020 is week 1/2020, corresponding week for 1/2021 is 2/2020 and so forth. Observation period for the first-wave and its control period is displayed on the left, spanning calendar weeks 10/2020—20/2020; observation period for the second-wave and its control period is displayed on the right, spanning calendar weeks 39/2020—8/2021. Weeks are only displayed, if data for complete weeks is available in both years (Covid and control period). Dotted black horizontal lines 1–5 indicate the following events: 1) Beginning of first lockdown in Germany at 03/22/2020; 2) End of first lockdown in Germany at 05/06/2020; 3) Beginning of "lockdown light" in Germany at 11/02/2020; 4) Beginning of the second strict lockdown in Germany at 12/16/2020; 5) first opening steps after second lockdown in Germany at 03/01/2021. Abbreviations used: pED = psychiatric emergency department; Covid = coronavirus disease

We observed 7.8% more pED presentations with SI (2019: *n* = 204, 2020: *n* = 220; *p* = 0.009), 38.2% more presentations with SP (2019: *n* = 60, 2020: *n* = 83; *p* = 0.003) and 89.5% more presentations after SA (2019: *n* = 19, 2020: *n* = 36; *p* = 0.004) during the first-wave compared to its control period. No significant changes in pED presentations with suicidality were observed during the second-wave and its control period. Figure 2 depicts suicidality rates in pED presentations per week during the observed time periods.

## Discussion

This research examines the impact of the Covid-19 pandemic on pED presentations with suicidality and further investigates differential effects across diagnostic categories. The picture arising from previous literature on pED presentations with suicidality during the Covid-19 pandemic is rather heterogenous: a study from Geneva reported similar results of an Odds ratio of 2.4 for suicidal behavior in 2020, when comparing a lockdown period in 2020 to the same period in 2016 [19]. In a study sample from Boston, an increase of SI was observed, with a maximal relative increase of 127.5% in one week compared to 2019[20]. In contrast, observations from other pEDs reported decreases in case numbers with SI from –3.4% [26] to –23% [44] and an even more pronounced decrease in SA [44]. Three studies found upward rebounds in post-lockdown periods [25–27], another found a downward rebound after loosening of restrictions[20]. A review by Giner et al. focusing on the first 6 months of the pandemic found no overall change in suicidal behavior, although pointing out the preliminary nature of most included papers and that long term consequences may have an effect at a later phase of the pandemic [45]. In our sample, the first-wave but not the second-wave was an independent risk factor for all three suicidality outcomes (Table 2). Several factors influencing suicidality during Covid-19 can be considered: McDowell et al. discuss stress triggered by lockdown measures, isolation and fear of infection as factors increasing suicidality [20], a view which is supported by reports of higher suicidality among those with self-reported strain during Covid-19 [46]. The Covid-19 containment measures differed between countries, which may explain some of the heterogeneity of findings on suicidality between studies. In Berlin, the lockdown during the first-wave came about with stricter containment rules than during the second-wave which started as “lockdown light” and gradually became stricter over time. One may speculate that the comparatively strict lockdown rules with consequent psychosocial strain during the first-wave led to an overall increase in suicidality in our sample. Another factor, which may play a role in the overall increase in suicidality is the reduction in inpatient treatment capacities that were implemented to prevent Covid-19 infections. This led to more strict indications for hospitalization and might have been an incentive for patients to report suicidality to increase their chances of inpatient treatment. The reduction may have influenced results regarding the first-wave, however inpatient treatment capacities were increased again after the first months of the pandemic and are therefore unlikely to play a role in the second-wave.

### Borderline personality disorder

In the SA model, the interaction effect of BPD and second-wave was estimated with a rate ratio of 7.128. This is the first evidence indicating an increase of suicidality during the Covid-19 period in this particularly vulnerable group. BPD was also time independently associated with a high risk for SI and SA (Table 2), which is congruent with literature on BPD [40]. However, the increase of suicidality from the already high baseline rate during the Covid-19 period cannot be found in previous literature. Research on BPD during the pandemic is too scarce to provide more than an indicative picture: a study from the outpatient clinic of the Department of Psychiatry at the Charité SHK, found that patients with PD were more often burdened by lockdown and hygiene measures and had less fear of a Covid-19 infection [47], which might lead to measures being perceived as too strict. Furthermore, the buffering effect of social support on impulsivity [48], a key trait of BPD [49], is likely to have decreased in times of social distancing and stay-at-home orders. A similar perspective is provided by a case report, which reported worsening of symptoms and a feeling of social rejection of a BPD patient [50]. A small study following 50 patients in a regular psychotherapeutic treatment in Spain reported no change in symptom severity, but could identify living alone as the most relevant predictor for a worse clinical outcome [51]. Preventing loneliness might thus be a key intervention point in this patient group. Further research needs to focus on BPD and the outcomes of patients within this group during the ongoing pandemic.

### Substance use disorders

Time dependent interaction effects estimated an elevated risk of SI and SP in patients with SUD during the second-wave. Research on SI during Covid-19 in the SUD cohort is not yet comprehensive enough to provide a complete picture, however, a pED study from Hannover found a similar increase of SI in patients with SUD [29]. Possible reasons for increased suicidality include a higher alcohol and drug use during the pandemic [52] and a higher number of relapses during the Covid-19 pandemic, which have been reported in a survey across SUD treatment providers in California [53]. Furthermore, increases in alcohol withdrawal related emergencies have been reported [54]. A large study in the US reported an increase in drug overdoses during the pandemic, possibly due to higher-risk types of drug use and worse access to harm-reduction measures [55]. The pandemic particularly seems to affect the life situation of patients with SUD, possibly leading to increased SI and SP as a form of severe SI. It may be speculated that an increase in SA by drug overdose also took place but was not seen in the current study, as SAs with SUD are often triaged as somatic cases and are admitted at intensive care units. Further research is needed to elucidate if there was also an increase in SA in patients with SUD.

### Depressive disorders

In all of our models (Table 2), DD is an important risk factor for suicidal outcomes, which is a similar result to previous findings on comorbidities of suicidality [56]. Yet, the interaction effect between DD and the first-wave estimated a decreased risk for SA (*p* = 0.018). This is remarkable, given that various studies observed increased depressive symptoms in the general population [1]. Patients with depression were less likely to attend the pED during the Covid-19 periods. This strong decline in overall numbers of pED presentations of patients with DD during the first Covid-19 wave was seen in our sample (supplements S3.2.1) as well as in other studies [19, 21, 57]. Patients with depression may have experienced strong fear of infection and possibly withdrew more than others from social contacts, leading to less self-motivated as well as less externally motivated pED presentations. The interaction effect indicating decreased risk for SA in patients with DD may also be influenced by the prominent decrease in pED attendances by patients with DD during the first-wave. Whether the discrepancy between possibly increased help needs and declined service utilization during the pandemic has led to higher suicide mortality should be addressed in future research.

### Schizophrenia and psychotic disorders

The role of patients with SPD during the pandemic is not yet fully understood. An increase in case numbers of patients with SPD has been described [58], which we also saw during the second-wave (supplements S3.2.2). Regarding symptom severity however, the picture is still unclear: in our sample, SPD was associated with decreased risk for SA during the second-wave (Table 2). In the literature, there are reports of better subjective wellbeing of this group during the first Covid-19 wave but coincidentally an important number of crisis admissions [47]. Others report an increase in persecutory delusions and visual hallucinations in this subgroup during the first-wave of Covid-19 [29]. When SA is considered as a clinical factor expressing severity, the reduction of risk for SA during the second-wave indicates rather a decrease in severity of pED presentations with patients with SPD during the second-wave of Covid-19. However, more research including other clinical characteristics of severity is necessary to test this assumption.

### Suicidality vs. suicides

Appleby et al. suggest that approximately a quarter of people who commit suicide have sought psychiatric help, including pEDs, in the year before death [59]. Predictions of increased suicides during the current pandemic were made based on models extrapolating suicide rates from unemployment and suicide rates of previous economic crises [60]. However, until now, studies examining suicide deaths found no overall change in suicide rates during the pandemic [16, 17, 61]. A rise in suicides may be specific to certain subgroups, e.g. in elderly women in Germany [17]. The current study, describing a pandemic-driven increase in suicidality in psychiatric patients in Berlin, during the first-wave overall and during the second-wave in patients with SUD and BPD, conveys no information about suicides and no studies exist at this point on this subject in Berlin. Future research should determine if there is a rise in suicides in Berlin during the pandemic. Ideally this research would also analyze subgroups of different age groups and across psychiatric diagnoses.

### Comparison of pED utilization

Weekly presentations to our pED decreased by 13.5% during the first-wave, which is in line with observations from other pEDs [19, 55, 62–75]. During the second-wave, the rate of weekly pED presentations did not change compared to its control period, but the number of patients decreased, which was reflected in a higher mean number of pED presentations per patient. This was not due to a small number of frequent attenders, but rather represents a trend in many patients, as this difference did not disappear when excluding those presenting more than 5 or 10 times (data not shown). This may be a belated effect of decreased accessibility to low threshold mental health care services such as patient group meetings, shifting help seeking to the pED. Another possibility would be that patients were less rapidly admitted than before because of a stricter admission policy due to reduced bed capacity for infection prevention reasons. To clarify this, more research is necessary.

### Other poisson regression results

SI showed time independent associations with diagnostic groups PD, DD, NSD, SUD, BMD and SPD. Regarding PD, DD, NSD and SUD, findings are in line with other studies on comorbidities of SI in psychiatric wards [76] and the general population [56]. BMD was not separately listed in research by Furnes et al. [76] or Nock et al. [30, 56], but evidence exists that BMD has the highest suicide risk among all psychiatric conditions[77]. It is important to point out the different outcome measures used, as pED presentations with suicidality are not equivalent to death by suicide (see above). The special role of BMD is not reflected in our Poisson regression results, as it only occurs as a minor risk factor in the SI model for the second-wave and its control period (Table 2). SPD showed a slightly elevated risk ratio in one SI model (Table 2), being in contrary to Furnes et al. [76] who showed a protective effect of SPD for SI. Since the effect of SPD estimated by our models is only small and occurs in only one of six models, it could represent a random finding. Other risk factors were being homeless, belonging to Covid-19 risk group, living in a residential psychiatric therapeutic environment as well as younger age. Being homeless is a known risk factor for SI [8]; belonging to Covid-19 risk group can be interpreted as a form of poor general health, which is also well established as a risk factor for SI [38, 78]. An elevated risk of those living in a residential psychiatric treatment environment seems logical, as it is part of social service for patients with mental illnesses in need of support with daily living and therefore likely associated with more severe, often chronic psychiatric conditions. Our observation that SI decrease with higher age is in line with previous studies on clinical populations [76].

Further risk factors for SP were PD, DD, NSD and SUD and are in line with previous research [56], although SP are not examined as often. Furthermore, being homeless and male gender are associated with an elevated risk of SP in our sample. A large cross-national study showed an elevated risk for females [56]. This difference could be influenced by the different underlying study population, as this study can only report for pED attendees (with a gender imbalance towards males), whereas Nock et al. followed an approach scoping at general populations [56].

## Strengths and limitations

A major strength of this study is the large sample size and the long observation periods. Based on thorough clinical documentation, it allowed us to investigate in a differentiated manner, which groups were at particular risk of increased suicidality. This is particularly relevant, as we were able to shed light onto the patients with BPD, which have been overlooked during the pandemic. Including the second-wave made a broader picture of developments possible, reaching further than a mere snapshot of the first few weeks of the global pandemic. Focusing on individual patients rather than on pED presentations on an event level allowed us to assess risk factors on an individual level as well as taking different service utilization patterns into account.

There are, however, also some limitations to be considered: our data is limited to one study site and may therefore be influenced by specific circumstances that limit generalizability. Furthermore, our study periods included only short post-lockdown periods and may thus miss rebound effects reported by other studies [25–27]. Also, control periods were restricted to 2019. Thus, some results might be accentuated by influences specific to 2019. The results of this study are based on qualitative exploration of suicidality by the examining psychiatric staff of our pED, who standardly explore suicidality. However, no standardized assessment instrument for exploring suicidality such as the Beck’s Scale for Suicide Ideation [79] has been used. As non-suicidal self-harm presentations are predominantly seen by the surgical department, they are not represented in our study population. This aspect should be accounted for in future research. A further limitation to our study is the lack of assessment regarding severity of psychiatric symptoms. As severity may affect suicidality, it should be assessed in future research. We were not able to investigate the effect of Covid-19 periods on suicidality in patients with BMD in our Poisson regression models, although we think this would be of importance. More research towards this group is necessary. We were not able to retrospectively differentiate between different forms of SA such as the differentiation between high- and low-risk suicide attempts as well as parasuicidal attempts. Future research should examine this.

## Conclusion

 In our sample suicidality generally increased during the first-wave of the Covid-19 pandemic across diagnoses compared to one year earlier. Patients with BPD were the only diagnostic subgroup displaying increased suicidality during the first-wave and second-wave of Covid-19 in comparison to the respective control periods and may therefore be at particular risk for suicidality. Patients with SUD also seem to be negatively affected by the pandemic, although only regarding SI and SP and only to a moderate extent, whereas BPD patients showed a drastically increased risk of SA. The outcomes for individuals with BPD and SUD during the ongoing pandemic should be targeted in future research.

## Supplementary Information

Below is the link to the electronic supplementary material.

Supplementary file1 (DOCX 52 KB)

## References

[1] Santomauro DF, Mantilla Herrera AM, Shadid J, Zheng P, Ashbaugh C, Pigott DM (2021). Global prevalence and burden of depressive and anxiety disorders in 204 countries and territories in 2020 due to the COVID-19 pandemic. Lancet.

[2] Pierce M, Hope H, Ford T, Hatch S, Hotopf M, John A (2020). Mental health before and during the COVID-19 pandemic: a longitudinal probability sample survey of the UK population. Lancet Psychiatr.

[3] Tsamakis K, Tsiptsios D, Ouranidis A, Mueller C, Schizas D, Terniotis C (2021). COVID-19 and its consequences on mental health (Review). Exp Ther Med.

[4] Iob E, Frank P, Steptoe A, Fancourt D (2020). Levels of severity of depressive symptoms among at-risk groups in the UK during the COVID-19 pandemic. JAMA Netw Open.

[5] Skoda EM, Bäuerle A, Schweda A, Dörrie N, Musche V, Hetkamp M (2021). Severely increased generalized anxiety, but not COVID-19-related fear in individuals with mental illnesses: A population based cross-sectional study in Germany. Int J Soc Psychiatr.

[6] de Beurs D, Bockting C, Kerkhof A, Scheepers F, O’Connor R, Penninx B (2021). A network perspective on suicidal behavior: understanding suicidality as a complex system. Suicide Life Threat Behav.

[7] Milner A, Page A, LaMontagne AD (2013). Long-term unemployment and suicide: a systematic review and meta-analysis. PLoS ONE.

[8] Ayano G, Tsegay L, Abraha M, Yohannes K (2019). Suicidal ideation and attempt among homeless people: a systematic review and meta-analysis. Psychiatr Q.

[9] Vandoros S, Kawachi I (2021). Economic uncertainty and suicide in the United States. Eur J Epidemiol.

[10] McClelland H, Evans JJ, Nowland R, Ferguson E, O'Connor RC (2020). Loneliness as a predictor of suicidal ideation and behaviour: a systematic review and meta-analysis of prospective studies. J Affect Disord.

[11] Howarth EJ, O'Connor DB, Panagioti M, Hodkinson A, Wilding S, Johnson J (2020). Are stressful life events prospectively associated with increased suicidal ideation and behaviour? A systematic review and meta-analysis. J Affect Disord.

[12] Liu S, Heinzel S, Haucke MN, Heinz A (2021). Increased psychological distress, loneliness, and unemployment in the spread of COVID-19 over 6 months in Germany. Medicina.

[13] O'Connor RC, Wetherall K, Cleare S, McClelland H, Melson AJ, Niedzwiedz CL (2021). Mental health and well-being during the COVID-19 pandemic: longitudinal analyses of adults in the UK COVID-19 mental health & wellbeing study. Br J Psychiatr.

[14] Radeloff D, Papsdorf R, Uhlig K, Vasilache A, Putnam K, von Klitzing K (2021). Trends in suicide rates during the COVID-19 pandemic restrictions in a major German city. Epidemiol Psychiatr Sci..

[15] Knudsen AKS, Stene-Larsen K, Gustavson K, Hotopf M, Kessler RC, Krokstad S (2021). Prevalence of mental disorders, suicidal ideation and suicides in the general population before and during the COVID-19 pandemic in Norway: A population-based repeated cross-sectional analysis. Lancet Reg Health Eur.

[16] Pirkis J, John A, Shin S, DelPozo-Banos M, Arya V, Analuisa-Aguilar P (2021). Suicide trends in the early months of the COVID-19 pandemic: an interrupted time-series analysis of preliminary data from 21 countries. Lancet Psychiatr.

[17] Radeloff D, Genuneit J, Bachmann CJ (2022) Suicides in Germany during the COVID-19 pandemic—an analysis based on data from 11 million inhabitants, 2017–2021. Dtsch Arztebl Int 119:502–3. 10.3238/arztebl.m2022.0198 10.3238/arztebl.m2022.0198 PMC9669323 36345581

[18] Schlump C, Thom J, Boender TS, Wagner B, Diercke M, Kocher T (2022). Using emergency department routine data for the surveillance of suicide attempts and psychiatric emergencies. Bundesgesundht Gesundh Gesundh.

[19] Ambrosetti J, Macheret L, Folliet A, Wullschleger A, Amerio A, Aguglia A (2021). Impact of the COVID-19 pandemic on psychiatric admissions to a large swiss emergency department: an observational study. Int J Environ Res Pub Health.

[20] McDowell MJ, Fry CE, Nisavic M, Grossman M, Masaki C, Sorg E (2021). Evaluating the association between COVID-19 and psychiatric presentations, suicidal ideation in an emergency department. PLoS ONE.

[21] Ferrando SJ, Klepacz L, Lynch S, Shahar S, Dornbush R, Smiley A (2021). Psychiatric emergencies during the height of the COVID-19 pandemic in the suburban New York City area. J Psychiatr Res.

[22] Rømer TB, Christensen RHB, Blomberg SN, Folke F, Christensen HC, Benros ME (2021). Psychiatric admissions, referrals, and suicidal behavior before and during the COVID-19 pandemic in Denmark: A Time-Trend Study. Acta Psychiatr Scand.

[23] Smalley CM, Malone DA, Meldon SW, Borden BL, Simon EL, Muir MR (2021). The impact of COVID-19 on suicidal ideation and alcohol presentations to emergency departments in a large healthcare system. Am J Emerg Med.

[24] Hernández-Calle D, Martínez-Alés G, Mediavilla R, Aguirre P, Rodríguez-Vega B, Bravo-Ortiz MF (2020). Trends in psychiatric emergency department visits due to suicidal ideation and suicide attempts during the COVID-19 pandemic in madrid. Spain J Clin Psychiatr.

[25] Ambrosetti J, Macheret L, Folliet A, Wullschleger A, Amerio A, Aguglia A (2021). Psychiatric emergency admissions during and after COVID-19 lockdown: short-term impact and long-term implications on mental health. BMC Psychiatr.

[26] Balestrieri M, Rucci P, Amendola D, Bonizzoni M, Cerveri G, Colli C (2021). Emergency psychiatric consultations during and after the COVID-19 Lockdown in Italy. Multicent Study Front Psychiatr.

[27] Boldrini T, Girardi P, Clerici M, Conca A, Creati C, Di Cicilia G (2021). Consequences of the COVID-19 pandemic on admissions to general hospital psychiatric wards in Italy: Reduced psychiatric hospitalizations and increased suicidality. Prog Neuropsychopharmacol Biol Psychiatr.

[28] Grossman MN, Fry CE, Sorg E, MacLean RL, Nisavic M, McDowell MJ (2021). Trends in suicidal ideation in an emergency department during COVID-19. J Psychosom Res.

[29] Seifert J, Meissner C, Birkenstock A, Bleich S, Toto S, Ihlefeld C (2021). Peripandemic psychiatric emergencies: impact of the COVID-19 pandemic on patients according to diagnostic subgroup. Eur Arch Psychiatr Clin Neurosci.

[30] Nock MK, Borges G, Bromet EJ, Cha CB, Kessler RC, Lee S (2008). Suicide and suicidal behavior. Epidemiol Rev.

[31] O'Loughlin S, Sherwood J (2005). A 20 year review of trends in deliberate self-harm in a British town, 1981–2000. Soc Psychiatr Psychiatr Epidemiol.

[32] Shah A (2007). The relationship between suicide rates and age: an analysis of multinational data from the World Health Organization. Int Psychogeriatr.

[33] Senatsverwaltung für Gesundheit Pflege und Gleichstellung: Coronavirus: Erster positiver Fall in Berlin bestätigt. https://www.berlin.de/sen/gpg/service/presse/2020/pressemitteilung.901388.php (2020). Accessed 06–22 2021.

[34] Landesamt für Gesundheit und Soziales: COVID-19 in Berlin, Fallzahlen und Indikatoren - Gesamtübersicht. https://www.berlin.de/lageso/gesundheit/infektionskrankheiten/corona/tabelle-indikatoren-gesamtuebersicht/ (2022). Accessed 01–06 2022.

[35] Kögel A. Erste Lockerung: Friseure öffnen am 1. März – das müssen Kunden wissen. Der Tagesspiegel2021.

[36] Robert-Koch-Institut: Epidemiologischer Steckbrief zu SARS-CoV-2 und COVID-19. https://www.rki.de/DE/Content/InfAZ/N/Neuartiges_Coronavirus/Steckbrief.html%3Bjsessionid=8939EA64CA73056801218A9D5D323C47.internet101?nn=13490888%23doc13776792bodyText15 (2021). Accessed 03.03. 2022.

[37] Bille-Brahe U (1982). Persons attempting suicide as clients in the Danish welfare system. Soc Psychiatr.

[38] Papadopoulou A, Efstathiou V, Yotsidi V, Pomini V, Michopoulos I, Markopoulou E (2021). Suicidal ideation during COVID-19 lockdown in Greece: prevalence in the community, risk and protective factors. Psychiatr Res.

[39] Gunderson JG, Ridolfi ME (2001). Borderline personality disorder. Ann N Y Acad Sci.

[40] Goodman M, Roiff T, Oakes AH, Paris J (2012). Suicidal risk and management in borderline personality disorder. Curr Psychiatr Rep.

[41] Isometsä E (2014). Suicidal behaviour in mood disorders—who, when, and why?. Can J Psychiatr.

[42] Guze SB, Robins E (1970). Suicide and primary affective disorders. Br J Psychiatr.

[43] Radomsky ED, Haas GL, Mann JJ, Sweeney JA (1999). Suicidal behavior in patients with schizophrenia and other psychotic disorders. Am J Psychiatr.

[44] Hörmann C, Bandli A, Bankwitz A, De Bardeci M, Rüesch A, De Araujo TV (2022). Suicidal ideations and suicide attempts prior to admission to a psychiatric hospital in the first six months of the COVID-19 pandemic: interrupted time-series analysis to estimate the impact of the lockdown and comparison of 2020 with 2019. BJPsych open..

[45] Giner L, Vera-Varela C, de la Vega D, Zelada GM, Guija JA (2022). Suicidal behavior in the first wave of the COVID-19 Pandemic. Curr Psychiatr Rep.

[46] Caballero-Domínguez CC, Jiménez-Villamizar MP, Campo-Arias A (2022). Suicide risk during the lockdown due to coronavirus disease (COVID-19) in Colombia. Death Stud.

[47] Winkler JG, Jalilzadeh Masah D, Moran JK, Bretz J, Tsagkas I, Goldschmidt T (2021). Psychological stress during the COVID-19 pandemic: consequences for psychiatric patients and therapeutic implications. Nervenarzt.

[48] Kleiman EM, Riskind JH, Schaefer KE, Weingarden H (2012). The moderating role of social support on the relationship between impulsivity and suicide risk. Crisis.

[49] McHugh C, Balaratnasingam S (2018). Impulsivity in personality disorders: current views and future directions. Current Opinion Psychiatr.

[50] Chong SC (2020). Psychological impact of coronavirus outbreak on borderline personality disorder from the perspective of mentalizing model: A case report. Asian J Psychiatr.

[51] Álvaro F, Navarro S, Palma C, Farriols N, Aliaga F, Solves L (2020). Clinical course and predictors in patients with borderline personality disorder during the COVID-19 outbreak: A 2.5-month naturalistic exploratory study in Spain. Psychiatr Res.

[52] Roberts A, Rogers J, Mason R, Siriwardena AN, Hogue T, Whitley GA (2021). Alcohol and other substance use during the COVID-19 pandemic: A systematic review. Drug Alcohol Depend.

[53] Henretty K, Padwa H, Treiman K, Gilbert M, Mark TL (2021). Impact of the coronavirus pandemic on substance use disorder treatment: findings from a survey of specialty providers in California. Subst Abuse.

[54] Schimmel J, Vargas-Torres C, Genes N, Probst MA, Manini AF (2021). Changes in alcohol-related hospital visits during COVID-19 in New York City. Addiction.

[55] Holland KM, Jones C, Vivolo-Kantor AM, Idaikkadar N, Zwald M, Hoots B (2021). Trends in US emergency department visits for mental health, overdose, and violence outcomes before and during the COVID-19 pandemic. JAMA Psychiat.

[56] Nock MK, Borges G, Bromet EJ, Alonso J, Angermeyer M, Beautrais A (2008). Cross-national prevalence and risk factors for suicidal ideation, plans and attempts. Br J Psychiatr.

[57] Capuzzi E, Di Brita C, Caldiroli A, Colmegna F, Nava R, Buoli M (2020). Psychiatric emergency care during Coronavirus 2019 (COVID 19) pandemic lockdown: results from a department of mental health and addiction of northern Italy. Psychiatr Res.

[58] Jagadheesan K, Danivas V, Itrat Q, Shekaran L, Lakra V (2021). A 6 month study on the pattern of emergency department presentations for schizophrenia and other psychotic disorders during COVID-19 lockdown. Psychiatry Res.

[59] Appleby L, Shaw J, Amos T, McDonnell R, Harris C, McCann K (1999). Suicide within 12 months of contact with mental health services: national clinical survey. BMJ.

[60] Kawohl W, Nordt C (2020). COVID-19, unemployment, and suicide. Lancet Psychiatr.

[61] Wollschläger D, Schmidtmann I, Blettner M, Ernst V, Fückel S, Caranci N (2021). Suicides during the COVID-19 pandemic 2020 compared to the years 2011–2019 in Rhineland-Palatinate (Germany) and Emilia-Romagna (Italy). Dtsch Arztebl Int.

[62] Gómez-Ramiro M, Fico G, Anmella G, Vázquez M, Sagué-Vilavella M, Hidalgo-Mazzei D (2021). Changing trends in psychiatric emergency service admissions during the COVID-19 outbreak: Report from a worldwide epicentre. J Affect Disord.

[63] Kratochvil D, Hill H, Moylan S (2021). The impact of Stage 3 COVID-19 lockdown on psychiatric presentations at a regional Victorian emergency department. Australas Psychiatr Bull Royal Aust N Z Coll Psychiatr.

[64] Dragovic M, Pascu V, Hall T, Ingram J, Waters F (2020). Emergency department mental health presentations before and during the COVID-19 outbreak in Western Australia. Australas Psychiatr Bull Royal Australian N Z Coll Psychiatr.

[65] Jagadheesan K, Danivas V, Itrat A, Lakra V (2021). Emergency department visits for psychiatric care during the first lockdown in Melbourne. Australas Psychiatr.

[66] Pignon B, Gourevitch R, Tebeka S, Dubertret C, Cardot H, Dauriac-Le Masson V (2020). Dramatic reduction of psychiatric emergency consultations during lockdown linked to COVID-19 in Paris and suburbs. Psychiatry Clin Neurosci.

[67] Montalbani B, Bargagna P, Mastrangelo M, Sarubbi S, Imbastaro B, De Luca GP (2021). The COVID-19 outbreak and subjects with mental disorders who presented to an Italian psychiatric emergency department. J Nerv Ment Dis.

[68] Beghi M, Ferrari S, Brandolini R, Casolaro I, Balestrieri M, Colli C (2021). Effects of lockdown on emergency room admissions for psychiatric evaluation: an observational study from 4 centres in Italy. Int J Psychiatr Clin Pract.

[69] Joyce LR, Richardson SK, McCombie A, Hamilton GJ, Ardagh MW (2021). Mental health presentations to Christchurch Hospital Emergency department during COVID-19 lockdown. Emerg Med Australas EMA.

[70] Gonçalves-Pinho M, Mota P, Ribeiro J, Macedo S, Freitas A (2021). The Impact of COVID-19 pandemic on psychiatric emergency department visits - a descriptive study. Psychiatry Q.

[71] Rodriguez-Jimenez R, Rentero D, Romero-Ferreiro V, García-Fernández L (2021). Impact of outbreak COVID-19 pandemic on psychiatry emergencies in Spain. Psychiatry Res.

[72] Yalçın M, Baş A, Bilici R, Özdemir Y, Beştepe EE, Kurnaz S (2021). Psychiatric emergency visit trends and characteristics in a mental health epicenter in Istanbul during COVID-19 lockdown. Soc Psychiatr Psychiatr Epidemiol.

[73] Butler M, Delvi A, Mujic F, Broad S, Pauli L, Pollak TA (2021). Reduced activity in an inpatient liaison psychiatry service during the first wave of the COVID-19 pandemic: comparison with 2019 data and characterization of the SARS-CoV-2 positive cohort. Front Psych.

[74] Hoyer C, Ebert A, Szabo K, Platten M, Meyer-Lindenberg A, Kranaster L (2021). Decreased utilization of mental health emergency service during the COVID-19 pandemic. Eur Arch Psychiatr Clin Neurosci.

[75] Håkansson A, Grudet C (2021). Decreasing psychiatric emergency visits, but stable addiction emergency visits, during COVID-19—A time series analysis 10 months into the pandemic. Front Psychiatr.

[76] Furnes D, Gjestad R, Rypdal K, Mehlum L, Hart S, Oedegaard KJ (2021). Suicidal and violent ideation in acute psychiatric inpatients: prevalence, co-occurrence, and associated characteristics. Suicide Life Threat Behav.

[77] Miller JN, Black DW (2020). Bipolar disorder and suicide: a review. Curr Psychiatr Rep.

[78] Cheah YK, Azahadi M, Phang SN, Abd Manaf NH (2018). Sociodemographic, lifestyle and health determinants of suicidal behaviour in Malaysia. Psychiatry Res.

[79] Kliem S, Lohmann A, Mößle T, Brähler E (2017). German Beck Scale for Suicide Ideation (BSS): psychometric properties from a representative population survey. BMC Psychiatry.

