# Supplementary material for: Suicidality in psychiatric emergency department situations during the first and the second wave of COVID-19 pandemic
Source: Eur Arch Psychiatry Clin Neurosci. 2022 Sep 7;273(2):311–23. doi: 10.1007/s00406-022-01486-6 (PMC9451117; doi:10.1007/s00406-022-01486-6)
Supplement: Supplementary file 1 — Supplementary file1 (DOCX 52 KB) [file 406_2022_1486_MOESM1_ESM.docx]

**Supplementary material**

**Contents:**

**S1 Criteria for Covid-19 risk group**

**S2 Composition of diagnostic categories**

**S3 Descriptive statistics**

**S3.1 Description of demographic and clinical characteristics of patients presenting to the psychiatric emergency department (pED)**

**S3.2 Descriptive statistics of patients attending the pED**

**S3.3 Description of suicidality across diagnostic categories**

**S3.4 Descriptive statistics of suicidality across diagnostic categories**

**S4 Goodness of fit parameters of regression models**

**S5 Sensitivity analysis: Negative binomial regression models**

**S1: Criteria for Covid-19 risk group**

For defining Covid-19 risk groups, classification of the Robert-Koch-Institut (RKI) was modified. The following criteria were used for defining the group, being at increased risk of adverse outcomes of Covid-19 infection. Note: criteria changed over time, as knowledge of the SARS-Cov-2 virus was growing over the course of the pandemic. We proceeded with what was known at the time of data extraction.

- Patients from 60 years of age and older
- Patients with chronic cardiac diseases (e.g. coronary heart disease, hypertension, etc.)
- Patients with chronic pulmonary diseases (e.g. COPD)
- Patients with chronic hepatic and renal diseases (e.g. liver cirrhosis)
- Patients with Diabetes mellitus
- Patients with Cancer and neoplastic diseases
- Patients with Immunodeficiency (e.g. AIDS, immunosuppression after organ transplantation)
- Patients with Adipositas

**S2: Composition of diagnostic categories**

Organic mental disorders (OMD): F00 – F09

Substance use disorders (SUD): F10 – F19

Not included: F17 nicotine/tobacco related substance use disorders

F1x.5 substance induced psychotic disorder

F1x.7 late-onset substance induced psychotic disorder

Schizophrenia and psychotic disorders (SPD): F20 – F29

F1x.5 substance induced psychotic disorder

F1x.7 late-onset substance induced psychotic disorder

Bipolar and manic disorders (BMD): F30 – F31

Depressive disorders (DD): F32 – F33

Neurotic, somatoform and

stress related disorders (NSD): F40 – F48

Personality disorders (PD): F60 – F62

Borderline personality disorder (BPD): F60.30 and F60.31

**S3.1: Description of demographic and clinical characteristics of patients presenting to the pED (patient-level)**

Supplementary table S4.2 displays the sociodemographic and clinical characteristics of patients presenting to the pED during the four observed periods. During the first-wave compared to its control-period (S4.2.1), patient numbers were 13.6% lower. During the second-wave compared to its control-period (S4.2.2), patient numbers were 10.4% lower. The mean number of presentations per patient did not differ between the first-wave and its control-period but was higher during the second-wave compared to its control-period (2019: 1.33 (0.917), 2020: 1.51 (1.421); p<.001; Cohen’s d: 0.153, supplementary table S4.2.2).

No differences were found regarding age, gender, homelessness, and Covid-19 risk group neither between the first-wave and its control period nor between the second-wave and its control-period. During the first-wave compared to its control-period, a higher number of patients living in residential psychiatric therapeutic environments presented to the pED (2019: n = 18 (2.5%), 2020: n = 30 (4.9%), p = 0.017). Regarding clinical characteristics, 43.3% less patients with PD (2019: n = 104 (14.2%), 2020: n = 59 (9.6%), p = 0.009) and 38.2% less patients with DD (2019: n = 157 (21.4%), 2020: n = 97 (15.7%), p = 0.008) presented to the pED during the first-wave compared to its control period. During the second-wave compared to its control-period, patients with SPD presented 5.9% more often (2019/2020: n = 372 (25.5%), 2020/2021: n = 394 (30.2%), p = 0.006) to the pED.

**S3.2: Descriptive statistics of patients attending the pED**

**S3.2.1: First wave**

|  | **control period (2019)** | **first-wave** | **difference** | **p-value** |  |
| --- | --- | --- | --- | --- | --- |
| **N total number of patients** | 733 | 617 | -15.8% |  |  |
| **Median age** | 39 years | 40 years | +1 year | 0.185 | Z = -1.325 |
| **Female gender (%)** | 314 (42.8%) | 235 (38.1%) | -25.2% | 0.077 |  |
| **mean number of presentations per patient (SD)** | 1.26 (0.770) | 1.29 (0.813) | +2.4% | 0.433 |  |
|  |  |  |  |  |  |
| **Living circumstances** |  |  |  |  |  |
| *Homeless patients (%)* | 60 (8.2%) | 69 (11.2%) | +15.0% | 0.062 |  |
| *Living in a residential psychiatric therapeutic environment (%)* | 18 (2.5%) | 30 (4.9%) | +66.7% | **0.017** |  |
|  |  |  |  |  |  |
| Covid-19 risk group (%) | 185 (25.2%) | 175 (28.4%) | -5.4% | 0.196 |  |
|  |  |  |  |  |  |
| **Diagnostic categories** |  |  |  |  |  |
| *Organic mental disorders (%)* | 35 (4.8%) | 33 (5.3%) | -5.7% | 0.631 |  |
| *Substance use disorders (%)* | 375 (51.2%) | 310 (50.2%) | -17.3% | 0.737 |  |
| *Schizophrenia and psychotic disorders (%)* | 206 (28.1%) | 198 (32.1%) | -3.9% | 0.111 |  |
| *Bipolar and manic disorders (%)* | 39 (5.3%) | 38 (6.2%) | -2.6% | 0.508 |  |
| *Depressive disorders (%)* | 157 (21.4%) | 97 (15.7%) | -38.2% | **0.008** |  |
| *Neurotic, somatoform and stress related disorders (%)* | 148 (20.2%) | 127 (20.6%) | -14.2% | 0.858 |  |
| *Personality disorders (%)* | 104 (14.2%) | 59 (9.6%) | -43.3% | **0.009** |  |
| *Borderline personality disorder (%)* | 62 (8.5%) | 36 (5.8%) | -41.9% | 0.064 |  |
| *Other personality disorders (%)* | 42 (5.7%) | 23 (3.7%) | -45.2% | 0.087 |  |

**S3.2.2: Second wave**

|  | **control period (2019/2020)** | **second-wave** | **difference** | **p-value** |  |
| --- | --- | --- | --- | --- | --- |
| **N total number of patients** | 1456 | 1304 | -10.4% |  |  |
| **Median age** | 39 years | 39 years | ±0 years | 0.434 | Z = -0.783 |
| **Female gender (%)** | 591 (40.6%) | 548 (42.1%) | -7.3% | 0.435 |  |
| **mean number of presentations per patient (SD)** | 1.33 (0.917) | 1.51 (1.421) | +13.5% | **<0.001** | Cohen's d = 0.153 (95% CI: 0.079 - 0.228) |
|  |  |  |  |  |  |
| **Living circumstances** |  |  |  |  |  |
| *Homeless patients (%)* | 146 (10.0%) | 161 (12.3%) | +10.3% | 0.053 |  |
| *Living in a residential psychiatric therapeutic environment (%)* | 88 (6.0%) | 103 (7.9%) | +17.0% | 0.055 |  |
|  |  |  |  |  |  |
| Covid-19 risk group (%) | 376 (25.8%) | 354 (27.1%) | -5.9% | 0.431 |  |
|  |  |  |  |  |  |
| **Diagnostic categories** |  |  |  |  |  |
| *Organic mental disorders (%)* | 92 (6.3%) | 81 (6.2%) | -12.0% | 0.908 |  |
| *Substance use disorders (%)* | 681 (46.8%) | 590 (45.2%) | -13.4% | 0.422 |  |
| *Schizophrenia and psychotic disorders (%)* | 372 (25.5%) | 394 (30.2%) | +5.9% | **0.006** |  |
| *Bipolar and manic disorders (%)* | 77 (5.3%) | 68 (5.2%) | -11.7% | 0.931 |  |
| *Depressive disorders (%)* | 296 (20.3%) | 241 (18.5%) | -18.6% | 0.221 |  |
| *Neurotic, somatoform and stress related disorders (%)* | 364 (25.0%) | 289 (22.2%) | -20.6% | 0.080 |  |
| *Personality disorders (%)* | 163 (11.2%) | 157 (12.0%) | -3.7% | 0.489 |  |
| *Borderline personality disorder (%)* | 110 (7.6%) | 97 (7.4%) | -11.8% | 0.908 |  |
| *Other personality disorders (%)* | 53 (3.6%) | 60 (4.6%) | +13.2% | 0.203 |  |

Comparison of demographic and clinical characteristics of patients attending the pED in corresponding time periods of the first-wave (S3.2.1) and the second-wave (S3.2.2). The difference column shows the change of patient numbers in the Covid-19 period compared to the corresponding control period in percentages. P-values are derived from chi²-tests, except for "median age" and "median number of presentations per patient", which were tested using the Mann-Whitney-U-test. "Mean number of presentations per patient" were not tested and are only displayed for description purposes. Covid-19 risk group is defined by a modified classification of the Robert-Koch-Institute, criteria can be found in supplementary material S2.2. "Living in a residential psychiatric therapeutic environment" is composed of patients living in therapeutic residential groups and living alone with psychiatric and/or social assistance. Abbreviations used: N = patient numbers; SHK = St. Hedwig Hospital; Covid-19 = coronavirus disease 2019; SD = standard deviation; pED = psychiatric emergency department

**S3.3 Description of suicidality across diagnostic categories**

Comparison of pED presentations with suicidality within diagnostic categories (Supplementary material S3.4) showed significant changes during the first-wave compared to its control-period in the group of NSD for pED presentations after SA (2019: n=0, 2020: n=7; p=.005) and in the group of PD for all three suicidality outcomes: a higher proportion of pED presentations with PD involved SI during the first-wave compared to its control period (2019: n=54 (38.0%), 2020: n=49 (59.0%); p=.002) and presentations with SP were more frequent by 33.3% in PD during the first-wave compared to its control-period (2019: n=18, 2020: n=24; p=.002), presentations after SA were 75% more frequent in PD during the first-wave compared to its control-period (2019: n=8, 2020: n=14; p=.006). Discriminating between presentations with BPD and other PD showed that suicidality was more frequent only in presentations with BPD during the first-wave compared to its control-period (SI 2019: n=36 (41.9%), 2020: n=36 (63.2%), p=.013; SP 2019: n=8, 2020: n=17, p=.001; SA 2019: n=5, 2020: n=10, p=.025), whereas in other PD no differences in suicidality occurred.

During the second-wave compared to its control-period, presentations with BPD showed 65% higher numbers of SP (2019: n=20, 2020: n=33; p=.04) and 240% more presentations after SA (2019: n=5; 2020: n=17; p=.007), whereas other PD displayed less SI (2019: n=49, 2020: n=41; p=.008) and less SP (2019: n=25, 2020: n=16; p=.014).

**S3.4 Descriptive statistics of suicidality across diagnostic categories**

**S3.4.1 First wave**

|  | **control period (2019)** | **first-wave** | **difference** | **p-value** |  |
| --- | --- | --- | --- | --- | --- |
|  |  |  |  |  |  |
| **Organic mental disorders** |  |  |  |  |  |
| *Suicidal ideation* | 2 (4.9%) | 4 (10.8%) | +100.0% | 0.415 | Fisher's exact test |
| *Suicide plans* | 0 (0%) | 2 (5.4%) | - | 0.222 | Fisher's exact test |
| *Suicide attempt* | 0 (0%) | 2 (5.4%) | - | 0.222 | Fisher's exact test |
| **Substance use disorders** |  |  |  |  |  |
| *Suicidal ideation* | 112 (24.1%) | 108 (27.1%) | -3.6% | 0.305 |  |
| *Suicide plans* | 36 (7.8%) | 41 (10.3%) | +13.8% | 0.188 |  |
| *Suicide attempt* | 12 (2.6%) | 18 (4.5%) | +50.0% | 0.119 |  |
| **Schizophrenia and psychotic disorders** |  |  |  |  |  |
| *Suicidal ideation* | 35 (12.8%) | 47 (17.3%) | +34.3% | 0.14 |  |
| *Suicide plans* | 8 (2.9%) | 10 (3.7%) | +25.0% | 0.62 |  |
| *Suicide attempt* | 3 (1.1%) | 7 (2.6%) | +133.3% | 0.221 | Fisher's exact test |
| **Bipolar and manic disorders** |  |  |  |  |  |
| *Suicidal ideation* | 6 (14.6%) | 12 (21.4%) | +100.0% | 0.395 |  |
| *Suicide plans* | 2 (4.9%) | 8 (14.5%) | +300.0% | 0.181 | Fisher's exact test |
| *Suicide attempt* | 1 (2.4%) | 1 (1.8%) | ±0.0% | 1 | Fisher's exact test |
| **Depressive disorders** |  |  |  |  |  |
| *Suicidal ideation* | 63 (38.0%) | 49 (46.2%) | -22.2% | 0.176 |  |
| *Suicide plans* | 20 (12.1%) | 19 (17.9%) | -5.0% | 0.184 |  |
| *Suicide attempt* | 10 (6.0%) | 7 (6.6%) | -30.0% | 0.847 |  |
| **Neurotic, somatoform and stress related disorders** |  |  |  |  |  |
| *Suicidal ideation* | 45 (27.6%) | 50 (34.2%) | +11.1% | 0.207 |  |
| *Suicide plans* | 14 (8.6%) | 16 (11.0%) | +14.3% | 0.47 |  |
| *Suicide attempt* | 0 (0%) | 7 (4.8%) | - | **0.005** | Fisher's exact test |
| **Personality disorders** |  |  |  |  |  |
| *Suicidal ideation* | 54 (38.0%) | 49 (59.0%) | -9.3% | **0.002** |  |
| *Suicide plans* | 18 (12.8%) | 24 (29.3%) | +33.3% | **0.002** |  |
| *Suicide attempt* | 8 (5.6%) | 14 (16.9%) | +75.0% | **0.006** |  |
| **Borderline personality disorder** |  |  |  |  |  |
| *Suicidal ideation* | 36 (41.9%) | 36 (63.2%) | ±0.0% | **0.013** |  |
| *Suicide plans* | 8 (9.4%) | 17 (30.4%) | +112.5% | **0.001** |  |
| *Suicide attempt* | 5 (5.8%) | 10 (17.5%) | +100.0% | **0.025** |  |
| **Other personality disorders** |  |  |  |  |  |
| *Suicidal ideation* | 18 (32.1%) | 13 (50.0%) | -27.8% | 0.121 |  |
| *Suicide plans* | 10 (17.9%) | 7 (26.9%) | -30.0% | 0.346 |  |
| *Suicide attempt* | 3 (5.4%) | 4 (15.4%) | +33.3% | 0.2 | Fisher's exact test |

**S3.4.2 Second wave**

|  | **control period (2019/2020)** | **second-wave** | **difference** | **p-value** |  |
| --- | --- | --- | --- | --- | --- |
|  |  |  |  |  |  |
| **Organic mental disorders** |  |  |  |  |  |
| *Suicidal ideation* | 17 (16.2%) | 16 (15.4%) | -5.9% | 0.873 |  |
| *Suicide plans* | 8 (7.7%) | 7 (6.7%) | -12.5% | 0.789 |  |
| *Suicide attempt* | 4 (3.8%) | 2 (1.9%) | -50.0% | 0.683 | Fisher's exact test |
| **Substance use disorders** |  |  |  |  |  |
| *Suicidal ideation* | 250 (26.6%) | 259 (27.8%) | +3.6% | 0.533 |  |
| *Suicide plans* | 103 (11.0%) | 126 (13.5%) | +23.5% | 0.089 |  |
| *Suicide attempt* | 41 (4.4%) | 47 (5.1%) | +14.6% | 0.48 |  |
| **Schizophrenia and psychotic disorders** |  |  |  |  |  |
| *Suicidal ideation* | 100 (19.3%) | 95 (15.8%) | -5.0% | 0.124 |  |
| *Suicide plans* | 33 (6.4%) | 44 (7.3%) | +33.3% | 0.531 |  |
| *Suicide attempt* | 17 (3.3%) | 13 (2.2%) | -23.5% | 0.250 |  |
| **Bipolar and manic disorders** |  |  |  |  |  |
| *Suicidal ideation* | 15 (15.6%) | 20 (21.3%) | +33.3% | 0.315 |  |
| *Suicide plans* | 5 (5.2%) | 8 (8.5%) | +60.0% | 0.367 |  |
| *Suicide attempt* | 1 (1.0%) | 0 (0%) | - | 1 | Fisher's exact test |
| **Depressive disorders** |  |  |  |  |  |
| *Suicidal ideation* | 146 (42.7%) | 122 (42.8%) | -16.4% | 0.976 |  |
| *Suicide plans* | 70 (20.5%) | 46 (16.1%) | -34.3% | 0.159 |  |
| *Suicide attempt* | 22 (6.4%) | 17 (6.0%) | -22.7% | 0.809 |  |
| **Neurotic, somatoform and stress related disorders** |  |  |  |  |  |
| *Suicidal ideation* | 130 (31.1%) | 114 (29.4%) | -12.3% | 0.596 |  |
| *Suicide plans* | 57 (13.6%) | 55 (14.2%) | -3.5% | 0.825 |  |
| *Suicide attempt* | 20 (4.8%) | 18 (4.6%) | -10.0% | 0.916 |  |
| **Personality disorders** |  |  |  |  |  |
| *Suicidal ideation* | 119 (50.4%) | 107 (42.0%) | -10.1% | 0.06 |  |
| *Suicide plans* | 45 (19.1%) | 49 (19.2%) | +8.9% | 0.985 |  |
| *Suicide attempt* | 11 (4.7%) | 22 (8.6%) | +100.0% | 0.082 |  |
| **Borderline personality disorder** |  |  |  |  |  |
| *Suicidal ideation* | 70 (46.7%) | 66 (44.9%) | -5.7% | 0.760 |  |
| *Suicide plans* | 20 (13.3%) | 33 (22.4%) | +65.0% | **0.04** |  |
| *Suicide attempt* | 5 (3.3%) | 17 (11.6%) | +240.0% | **0.007** |  |
| **Other personality disorders** |  |  |  |  |  |
| *Suicidal ideation* | 49 (57.0%) | 41 (38.0%) | -16.3% | **0.008** |  |
| *Suicide plans* | 25 (29.4%) | 16 (14.8%) | -36.0% | **0.014** |  |
| *Suicide attempt* | 6 (7.1%) | 5 (4.6%) | -16.7% | 0.54 | Fisher's exact test |

**S4: Goodness of fit parameters of regression models**

Goodness of fit parameters for Poisson-regression models (primary analysis, Table 3), alternative negative binomial regression models (displayed as sensitivity analysis, supplementary material S8)

Suicidal ideation during the first-wave:

|  | Poisson, primary analysis | Negative binomial (1) |
| --- | --- | --- |
| value/df | 0.753 | 0.575 |
| Pearson Chi² value/df | 0.911 | 0.711 |
| AIC | 1809.146 | 1879.826 |

Suicidal ideation during the second-wave:

|  | Poisson, primary analysis | Negative binomial (1) |
| --- | --- | --- |
| value/df | 0.823 | 0.613 |
| Pearson Chi² value/df | 0.995 | 0.753 |
| AIC | 3986.748 | 4095.220 |

Suicidal plans during the first-wave:

|  | Poisson, primary analysis | Negative binomial (1) |
| --- | --- | --- |
| value/df | 0.428 | 0.358 |
| Pearson Chi² value/df | 0.981 | 0.891 |
| AIC | 863.659 | 871.641 |

Suicidal plans during the second-wave:

|  | Poisson, primary analysis | Negative binomial (1) |
| --- | --- | --- |
| value/df | 0.563 | 0.456 |
| Pearson Chi² value/df | 1.124 | 0.990 |
| AIC | 2286.426 | 2272.870 |

Suicide attempts during the first-wave:

|  | Poisson, primary analysis | Negative binomial (1) |
| --- | --- | --- |
| value/df | 0.188 | 0.165 |
| Pearson Chi² value/df | 0.891 | 0.851 |
| AIC | 379.347 | 383.557 |

Suicide attempts during the second-wave:

|  | Poisson, primary analysis | Negative binomial (1) |
| --- | --- | --- |
| value/df | 0.271 | 0.237 |
| Pearson Chi² value/df | 0.995 | 0.951 |
| AIC | 1028.803 | 1032.643 |

**S5: Sensitivity analysis: Negative binomial regression models**

|  | **Suicidal ideation (SI)** |  |
| --- | --- | --- |
| **Model type** | **Negative binomial (1)** |  |
|  | **first-wave and its control period** |  |
|  | RateRatio (95% CI) | p-value |
| *Covid-19 (vs. Control)* | 1.608 (1.020 - 2.534) | **0.041** |
| **Interaction effects (time dependent)** |  |  |
| *Borderline personality disorder by Covid-19* | 1.406 (0.710 - 2.784) | 0.328 |
| *Substance use disorders by Covid-19* | 0.751 (0.468 - 1.205) | 0.235 |
| *Depressive disorders by Covid-19* | 0.878 (0.498 - 1.550) | 0.655 |
| *Schizophrenia and psychotic disorders by Covid-19* | 1.030 (0.578 - 1.836) | 0.919 |
| **Diagnostic categories (time independent)** |  |  |
| *Organic mental disorders* | 0.635 (0.314 - 1.287) | 0.208 |
| *Substance use disorder* | 1.548 (1.092 - 2.195) | 0.014 |
| *Schizophrenia and psychotic disorders* | 0.854 (0.553 - 1.319) | 0.477 |
| *Bipolar and manic disorders* | 1.027 (0.590 - 1.787) | 0.925 |
| *Depressive disorders* | 2.208 (1.499 - 3.252) | **<0.001** |
| *Neurotic, somatoform and stress related disorders* | 1.429 (1.070 - 1.908) | **0.016** |
| *Borderline personality disorder* | 2.452 (1.519 - 3.959) | **<0.001** |
| *Other personality disorders* | 1.774 (1.121 - 2.809) | **0.014** |
| **Sociodemographic risk factors (time independent)** |  |  |
| *Male* | 1.002 (0.782 - 1.283) | 0.989 |
| *Age* | 0.992 (0.983 - 1.001) | 0.086 |
| *Living in residential psychiatric therapeutic environment* | 1.524 (0.885 - 2.625) | 0.129 |
| *Homeless* | 1.435 (0.997 - 2.065) | 0.052 |
| *Covid-19 risk group* | 1.191 (0.881 - 1.611) | 0.256 |

|  | **Suicidal ideation (SI)** |  |
| --- | --- | --- |
| **Model type** | **Negative binomial (1)** |  |
|  | **second-wave and its control period** |  |
|  | RateRatio (95% CI) | p-value |
| *Covid-19 (vs. Control)* | 0.994 (0.745 - 1.325) | 0.965 |
| **Interaction effects (time dependent)** |  |  |
| *Borderline personality disorder by Covid-19* | 0.874 (0.550 - 1.390) | 0.571 |
| *Substance use disorders by Covid-19* | 1.320 (0.967 - 1.801) | 0.080 |
| *Depressive disorders by Covid-19* | 1.002 (0.693 - 1.450) | 0.990 |
| *Schizophrenia and psychotic disorders by Covid-19* | 0.731 (0.501 - 1.066) | 0.103 |
| **Diagnostic categories (time independent)** |  |  |
| *Organic mental disorders* | 1.029 (0.678 - 1.560) | 0.894 |
| *Substance use disorder* | 1.279 (1.021 - 1.601) | **0.032** |
| *Schizophrenia and psychotic disorders* | 1.284 (0.974 - 1.692) | 0.076 |
| *Bipolar and manic disorders* | 1.368 (0.957 - 1.955) | 0.086 |
| *Depressive disorders* | 2.271 (1.756 - 2.937) | **<0.001** |
| *Neurotic, somatoform and stress related disorders* | 1.541 (1.276 - 1.861) | **<0.001** |
| *Borderline personality disorder* | 2.962 (2.129 - 4.121) | **<0.001** |
| *Other personality disorders* | 2.091 (1.541 - 2.836) | **<0.001** |
| **Sociodemographic risk factors (time independent)** |  |  |
| *Male* | 1.150 (0.973 - 1.358) | 0.102 |
| *Age* | 0.991 (0.985 - 0.997) | **0.005** |
| *Living in residential psychiatric therapeutic environment* | 1.355 (1.022 - 1.798) | **0.035** |
| *Homeless* | 1.483 (1.180 - 1.865) | **0.001** |
| *Covid-19 risk group* | 1.443 (1.167 - 1.785) | **0.001** |

|  | **Suicide plans (SP)** |  |
| --- | --- | --- |
| **Model type** | **Negative binomial (1)** |  |
|  | **first-wave and its control period** |  |
|  | RateRatio (95% CI) | p-value |
| *Covid-19 (vs. Control)* | 2.763 (1.299 - 5.881) | **0.008** |
| **Interaction effects (time dependent)** |  |  |
| *Borderline personality disorder by Covid-19* | 1.790 (0.611 - 5.240) | 0.288 |
| *Substance use disorders by Covid-19* | 0.537 (0.244 - 1.181) | 0.122 |
| *Depressive disorders by Covid-19* | 0.669 (0.277 - 1.613) | 0.371 |
| *Schizophrenia and psychotic disorders by Covid-19* | 0.770 (0.269 - 2.201) | 0.625 |
| **Diagnostic categories (time independent)** |  |  |
| *Organic mental disorders* | 0.319 (0.073 - 1.396) | 0.129 |
| *Substance use disorder* | 1.726 (0.930 - 3.201) | 0.083 |
| *Schizophrenia and psychotic disorders* | 0.651 (0.278 - 1.475) | 0.304 |
| *Bipolar and manic disorders* | 1.626 (0.770 - 3.433) | 0.203 |
| *Depressive disorders* | 2.601 (1.370 - 4.939) | **0.003** |
| *Neurotic, somatoform and stress related disorders* | 1.086 (0.666 - 1.772) | 0.741 |
| *Borderline personality disorder* | 1.952 (0.841 - 4.530) | 0.119 |
| *Other personality disorders* | 2.561 (1.351 - 4.855) | **0.004** |
| **Sociodemographic risk factors (time independent)** |  |  |
| *Male* | 1.481 (0.984 - 2.230) | 0.060 |
| *Age* | 0.996 (0.981 - 1.011) | 0.561 |
| *Living in residential psychiatric therapeutic environment* | 1.095 (0.435 - 2.754) | 0.848 |
| *Homeless* | 1.642 (0.941 - 2.868) | 0.081 |
| *Covid-19 risk group* | 1.458 (0.913 - 2.330) | 0.114 |

|  | **Suicide plans (SP)** |  |
| --- | --- | --- |
| **Model type** | **Negative binomial (1)** |  |
|  | **second-wave and its control period** |  |
|  | RateRatio (95% CI) | p-value |
| *Covid-19 (vs. Control)* | 0.859 (0.566 - 1.306) | 0.477 |
| **Interaction effects (time dependent)** |  |  |
| *Borderline personality disorder by Covid-19* | 1.678 (0.848 - 3.317) | 0.137 |
| *Substance use disorders by Covid-19* | 1.701 (1.083 - 2.672) | **0.021** |
| *Depressive disorders by Covid-19* | 0.836 (0.500 - 1.399) | 0.496 |
| *Schizophrenia and psychotic disorders by Covid-19* | 1.028 (0.586 - 1.805) | 0.923 |
| **Diagnostic categories (time independent)** |  |  |
| *Organic mental disorders* | 0.978 (0.540 - 1.772) | 0.942 |
| *Substance use disorder* | 1.167 (0.838 - 1.625) | 0.360 |
| *Schizophrenia and psychotic disorders* | 0.929 (0.603 - 1.431) | 0.739 |
| *Bipolar and manic disorders* | 1.280 (0.755 - 2.169) | 0.360 |
| *Depressive disorders* | 2.431 (1.705 - 3.468) | **<0.001** |
| *Neurotic, somatoform and stress related disorders* | 1.573 (1.203 - 2.056) | **0.001** |
| *Borderline personality disorder* | 1.700 (0.996 - 2.900) | 0.052 |
| *Other personality disorders* | 1.972 (1.298 - 2.996) | **0.001** |
| **Sociodemographic risk factors (time independent)** |  |  |
| *Male* | 1.400 (1.096 - 1.788) | **0.007** |
| *Age* | 0.997(0.988 - 1.006) | 0.507 |
| *Living in residential psychiatric therapeutic environment* | 1.093 (0.707 - 1.689) | 0.689 |
| *Homeless* | 1.577 (1.150 - 2.162) | **0.005** |
| *Covid-19 risk group* | 1.271 (0.936 - 1.726) | 0.124 |

|  | **Suicide attempt (SA)** |  |
| --- | --- | --- |
| **Model type** | **Negative binomial (1)** |  |
|  | **first-wave and its control period** |  |
|  | RateRatio (95% CI) | p-value |
| *Covid-19 (vs. Control)* | 10.185 (2.096 - 49.487) | **0.004** |
| **Interaction effects (time dependent)** |  |  |
| *Borderline personality disorder by Covid-19* | 1.219 (0.248 - 5.979) | 0.808 |
| *Substance use disorders by Covid-19* | 0.398 (0.100 - 1.579) | 0.190 |
| *Depressive disorders by Covid-19* | 0.167 (0.035 - 0.810) | **0.026** |
| ***Schizophrenia and psychotic disorders by Covid-19*** | 0.457 (0.081 - 2.585) | 0.376 |
| Diagnostic categories (time independent) |  |  |
| *Organic mental disorders* | 0.815 (0.156 - 4.248) | 0.808 |
| *Substance use disorder* | 1.730 (0.566 - 5.284) | 0.336 |
| *Schizophrenia and psychotic disorders* | 1.748 (0.409 - 7.472) | 0.451 |
| *Bipolar and manic disorders* | 0.498 (0.064 - 3.897) | 0.507 |
| *Depressive disorders* | 9.429 (2.827 - 31.443) | **<0.001** |
| *Neurotic, somatoform and stress related disorders* | 0.397 (0.133 - 1.185) | 0.098 |
| *Borderline personality disorder* | 6.660 (1.776 - 24.983) | **0.005** |
| *Other personality disorders* | 3.252 (1.139 - 9.282) | **0.028** |
| **Sociodemographic risk factors (time independent)** |  |  |
| *Male* | 2.429 (1.177 - 5.014) | **0.016** |
| *Age* | 1.009 (0.984 - 1.035) | 0.474 |
| *Living in residential psychiatric therapeutic environment* | 0.567 (0.073 - 4.391) | 0.587 |
| *Homeless* | 1.330 (0.503 - 3.516) | 0.565 |
| *Covid-19 risk group* | 1.247 (0.566 - 2.748) | 0.585 |

|  | **Suicide attempt (SA)** |  |
| --- | --- | --- |
| **Model type** | **Negative binomial (1)** |  |
|  | **second-wave and its control period** |  |
|  | RateRatio (95% CI) | p-value |
| *Covid-19 (vs. Control)* | 1.135 (0.575 - 2.244) | 0.715 |
| **Interaction effects (time dependent)** |  |  |
| *Borderline personality disorder by Covid-19* | 7.076 (1.472 - 34.005) | **0.015** |
| *Substance use disorders by Covid-19* | 1.295 (0.619 - 2.710) | 0.493 |
| *Depressive disorders by Covid-19* | 0.667 (0.286 - 1.557) | 0.350 |
| *Schizophrenia and psychotic disorders by Covid-19* | 0.347 (0.122 - 0.984) | **0.047** |
| **Diagnostic categories (time independent)** |  |  |
| *Organic mental disorders* | 1.175 (0.471 - 2.929) | 0.729 |
| *Substance use disorder* | 1.240 (0.713 - 2.157) | 0.446 |
| *Schizophrenia and psychotic disorders* | 1.028 (0.522 - 2.023) | 0.937 |
| *Bipolar and manic disorders* | 0.357 (0.085 - 1.494) | 0.158 |
| *Depressive disorders* | 2.004 (1.103 - 3.644) | **0.023** |
| *Neurotic, somatoform and stress related disorders* | 1.199 (0.762 - 1.885) | 0.432 |
| *Borderline personality disorder* | 0.402 (0.095 - 1.712) | 0.218 |
| *Other personality disorders* | 1.441 (0.677 - 3.067) | 0.343 |
| **Sociodemographic risk factors (time independent)** |  |  |
| *Male* | 0.972 (0.653 - 1.446) | 0.887 |
| *Age* | 0.994 (0.980 - 1.009) | 0.461 |
| *Living in residential psychiatric therapeutic environment* | 0.984 (0.464 - 2.089) | 0.967 |
| *Homeless* | 1.166 (0.657 - 2.068) | 0.599 |
| *Covid-19 risk group* | 1.090 (0.648 - 1.835) | 0.745 |
